# Measuring Mental Workload with EEG+fNIRS

**DOI:** 10.3389/fnhum.2017.00359

**Published:** 2017-07-14

**Authors:** Haleh Aghajani, Marc Garbey, Ahmet Omurtag

**Affiliations:** ^1^Department of Biomedical Engineering, University of Houston Houston, TX, United States; ^2^Center for Computational Surgery, Department of Surgery, Research Institute, Houston Methodist Houston, TX, United States

**Keywords:** functional near-infrared spectroscopy (fNIRS), electroencephalography (EEG), human mental workload, cognitive state monitoring, *n*-back, multi-modal brain recording, machine learning

## Abstract

We studied the capability of a Hybrid functional neuroimaging technique to quantify human mental workload (MWL). We have used electroencephalography (EEG) and functional near-infrared spectroscopy (fNIRS) as imaging modalities with 17 healthy subjects performing the letter *n*-back task, a standard experimental paradigm related to working memory (WM). The level of MWL was parametrically changed by variation of *n* from 0 to 3. Nineteen EEG channels were covering the whole-head and 19 fNIRS channels were located on the forehead to cover the most dominant brain region involved in WM. Grand block averaging of recorded signals revealed specific behaviors of oxygenated-hemoglobin level during changes in the level of MWL. A machine learning approach has been utilized for detection of the level of MWL. We extracted different features from EEG, fNIRS, and EEG+fNIRS signals as the biomarkers of MWL and fed them to a linear support vector machine (SVM) as train and test sets. These features were selected based on their sensitivity to the changes in the level of MWL according to the literature. We introduced a new category of features within fNIRS and EEG+fNIRS systems. In addition, the performance level of each feature category was systematically assessed. We also assessed the effect of number of features and window size in classification performance. SVM classifier used in order to discriminate between different combinations of cognitive states from binary- and multi-class states. In addition to the cross-validated performance level of the classifier other metrics such as sensitivity, specificity, and predictive values were calculated for a comprehensive assessment of the classification system. The Hybrid (EEG+fNIRS) system had an accuracy that was significantly higher than that of either EEG or fNIRS. Our results suggest that EEG+fNIRS features combined with a classifier are capable of robustly discriminating among various levels of MWL. Results suggest that EEG+fNIRS should be preferred to only EEG or fNIRS, in developing passive BCIs and other applications which need to monitor users' MWL.

## Introduction

Mental workload (MWL) affects people who are interacting with computers and other devices. The use of technology in everyday life may impose high cognitive demands as users navigate complex interfaces. Mental overload may compromise users' performance and sometimes safety, by increasing error rates and engendering fatigue, decline in motivation, higher reaction times (Xie and Salvendy, 2000; Young and Stanton, 2002), and neglect of critical information, known as cognitive tunneling (Thomas and Wickens, 2001; Dixon et al., 2013; Dehais et al., 2014). Taking into account the users' cognitive characteristics and limitations are thus critical in improving the design of human-machine interfaces (BMI) and for operating them efficiently by installing adaptive features that can respond to changes in the MWL (Kaber et al., 2000; Parasuraman and Wilson, 2008; Gagnon et al., 2012).

MWL has been defined as the proportion of the human operator's mental capabilities that is occupied during the performance of a given task (Boff et al., 1994). According to the prevalent Multiple Resources theory (Navon and Gopher, 1979; Wickens, 2002), performing different tasks requires a subject to tap into a set of separate resources, which are limited in capacity and distributable among tasks (Horrey and Wickens, 2003). In general, these resources can be categorized among four dimensions: processing stage (perception or cognition vs. response), perceptual modality (visual vs. auditory), visual channel (focal vs. ambient), and processing code (verbal vs. spatial; Wickens, 2002; Horrey and Wickens, 2003). Based on the Multiple Resources theory, equal resource demands between two tasks that both recruit one level of a given dimension will interfere with each other more than two tasks that recruit separate levels on the dimension (Wickens, 2002), and may create bottlenecks and consequent decrements in performance. Similar conclusions have been reached in the areas of aviation (Stanney and Hale, 2012; Causse and Matton, 2014; Durantin et al., 2014), education (Palmer and Kobus, 2007; Spüler et al., 2016), and a variety of clinical situations (Carswell et al., 2005; Stefanidis et al., 2007; Prabhu et al., 2010; Yurko et al., 2010; Byrne, 2013; Guru et al., 2015). In the case of driving while having a phone conversation, in addition to the interference of resources the “engagement phenomenon” also controls the outcome of multi-tasking scenario. This happens when one of the tasks attracts so much attention that the advantage of separate resource demand would be eliminated (Strayer and Johnston, 2001; Strayer and Drews, 2007).

MWL is a construct that arises from the interaction of the properties of a task, the environment in which it is performed, and the characteristics of the human operator performing it (Longo, 2016). Task properties include the difficulty and monotony of the task and the types of resources that it engages. The environment may contain various degrees of distraction and noise. The subject characteristics involve training and expertise as well as changing levels of fatigue, motivation, and vigilance. Thus, the MWL can be systematically adjusted by tuning a subset of these variables while controlling for the rest.

Methods of determining MWL fall into three broad categories: (1) Self-reporting and subjective ratings using standard questionnaires such as the NASA-TLX (Hart and Staveland, 1988); (2) Behavioral measures, such as primary- and secondary-task performance; and (3) Measures based on the physiology of the user, including heart rate variability, oculomotor activity, pupillometry, electromyography, galvanic skin response, and brain activity (Xiao et al., 2005; Wickens, 2008; Sahayadhas et al., 2012). Self-reporting and behavioral based information tends to be delayed, sporadic, and intrusive to obtain. Performance based information, in addition, can be misleading since multiple degrees of MWL may accompany the same level of performance (Yurko et al., 2010). Physiological measures, on the other hand, do not require overt behavior, can be arranged to have little or no interference with task execution, and can supply information continuously without significant delay. Progresses in miniaturization and wireless technology have amplified these advantages of physiological measures (Sahayadhas et al., 2012).

Most studies of MWL based on brain function have utilized electroencephalography (EEG), following a large number of studies using EEG for developing BMI (Wolpaw and Wolpaw, 2012). Functional near-infrared spectroscopy (fNIRS) as a newer modality have shown promising capabilities in BMI applications for discrimination of different motor tasks (Naseer and Hong, 2013) or decoding subjects' binary decisions (Naseer et al., 2014). The relationship between MWL and central nervous system activity is well-established (McBride and Schmorrow, 2005). BMIs that do not attempt to directly control a device but modulate its user interface based on real-time user status are referred as passive BMIs (Gateau et al., 2015). In such applications, of paramount importance. Recently multi-modal techniques utilizing concurrent EEG and fNIRS have gained popularity due to their relatively richer information content (Hirshfield et al., 2009; Liu Y. et al., 2013; Liu T. et al., 2013; Keles et al., 2014b; Aghajani and Omurtag, 2016; Buccino et al., 2016; Omurtag et al., 2017). Available evidence indicates that brain activity measures of MWL are more informative than ocular or peripheral physiology measures (Hogervorst et al., 2014).

Concurrent EEG and fNIRS, which we refer to as EEG+fNIRS, is promising as a practical technique that is more accurate than the individual modalities alone. fNIRS provides information that is complementary to EEG, by measuring the changes in cerebral blood flow (CBF) and related hemoglobin concentrations through near-infrared light source/detectors on the scalp. It is comparable to EEG in portability. fNIRS does not have electromyographic (EMG) and blink artifacts and its signal closely correlates with the blood oxygen level dependent (BOLD) signal from functional magnetic resonance imaging (fMRI; Strangman et al., 2002; Huppert et al., 2006), which is a gold-standard for measuring cerebral hemodynamics. In addition to the advantages of pooling different types of signals, EEG+fNIRS offers new types of features, ultimately based on neurovascular coupling (NVC), the cascade of processes by which neural activity modulates local blood flow and oxygenation, and NVC related features are not resolvable by a uni-modal signal sensitive to only neural activity (e.g., EEG) or only hemodynamics (e.g., BOLD).

Working memory (WM) is a brain system that provides transient holding and processing of the information necessary for complex cognitive tasks (Baddeley, 2003). It has been investigated in previous functional neuroimaging studies, which identified the prefrontal cortex (PFC) as the most relevant area of activation (Cohen et al., 1997; Smith and Jonides, 1997; Hoshi et al., 2003; Owen et al., 2005). MWL detection using WM load as an experimental paradigm has been studied using EEG (Berka et al., 2007; Dornhege et al., 2007; Grimes et al., 2008; Brouwer et al., 2012), fNIRS (Hoshi et al., 2003; Izzetoglu et al., 2003; Ayaz et al., 2012; Durantin et al., 2014; Herff et al., 2014), and concurrent EEG and fNIRS (Hirshfield et al., 2009; Coffey et al., 2012). We have previously shown that aspects of NVC are characterizable by EEG+fNIRS, by taking advantage of the synergistic interaction between the modalities (Keles et al., 2016). The potential of EEG+fNIRS for active BCIs has recently been investigated (Fazli et al., 2012; Liu Y. et al., 2013; Tomita et al., 2014; Buccino et al., 2016). In this study, we built on this work to explore the unique properties of EEG+fNIRS for MWL detection.

The *n*-back task was introduced by Kirchner (1958). *n*-back is a continues-performance task for measurement of WM capacity, which has been used frequently in the field of cognitive neuroscience. Gevins (Gevins et al., 1997, 1998; Smith and Gevins, 2005a) and Smith (Smith and Gevins, 2005b) showed during high task-load conditions EEG theta activity increases in the frontal midline and alpha activity attenuates during the performance of an *n*-back task. In addition, fNIRS revealed WM load while performing *n*-back task activates PFC (Ayaz et al., 2012; Sato et al., 2013; Fishburn et al., 2014; Herff et al., 2014; Mandrick et al., 2016b). The *n*-back task engages WM and becomes more demanding as the value of *n* increases. We have therefore used the *n*-back task as our experimental paradigm with *n* ranging from 0 to 3, allowing us to tune the task difficulty. Our study maintained all other conditions constant by employing only healthy adult volunteers with no previous experience with this task, all performing under the same laboratory conditions with no distractions or additional activities. Our experimental design is consistent with numerous BMI studies that take WM load as a proxy for MWL (Berka et al., 2007; Grimes et al., 2008; Coffey et al., 2012; Herff et al., 2014).

The aim of this study was, first, to introduce and validate a state of the art EEG+fNIRS set up in a single headpiece. Since PFC is the main region of interest in WM load detection (Owen et al., 2005), our design had the advantage of frontal lobe coverage by fNIRS. We had whole-head coverage by EEG. We used the term whole-head to refer to the fact that we placed EEG electrodes at all (except frontopolar) standard 10–20 sites bilaterally covering the frontal, central, temporal, parietal, and occipital areas. Having fNIRS optodes on the forehead not only improved the quality of acquired signal but also reduced the preparation time. The second aim of this study was to develop EEG+fNIRS measures that discriminate among levels of MWL and show that they are promising for the practical and accurate quantification of MWL in real-world settings. We developed such measures by extracting EEG, fNIRS, and Hybrid (EEG+fNIRS) based features from the full set of signals. Most discriminating features were selected and fed into support vector machines (SVM) to perform binary or multi-class classification. The handful of EEG+fNIRS studies currently available (Coffey et al., 2012; Putze et al., 2014; Buccino et al., 2016) have not systematically quantified the performance of subsets of a Hybrid system and its features contribute to the accuracy of classification. Therefore, the third aim of this study was to rigorously compare the performance of uni-modal and Hybrid systems.

## Methods

### Subjects

Seventeen healthy volunteers (16 males, 1 female) with a mean age of 26.2 and standard deviation of 7.7 years from University of Houston students or employees participated in the experiment. The experimental procedures involving human subjects described in this paper were approved by the Institutional Review Board of the University of Houston. The participants gave written informed consent prior to the experiments and were compensated for their effort by being given a gift card from a major retailer. During the performance of the verbal *n*-back task, target letters should be detected by the operator by means of pressing Space button on the keyboard. All subjects were right-handed and used their dominant hand for performing the experiment. This will reduce the variability of brain signals based on the motor function through all subjects. None of the subjects had ever taken part in an *n*-back study, thus no training effects were expected.

### Experimental design

**Figure 1:**
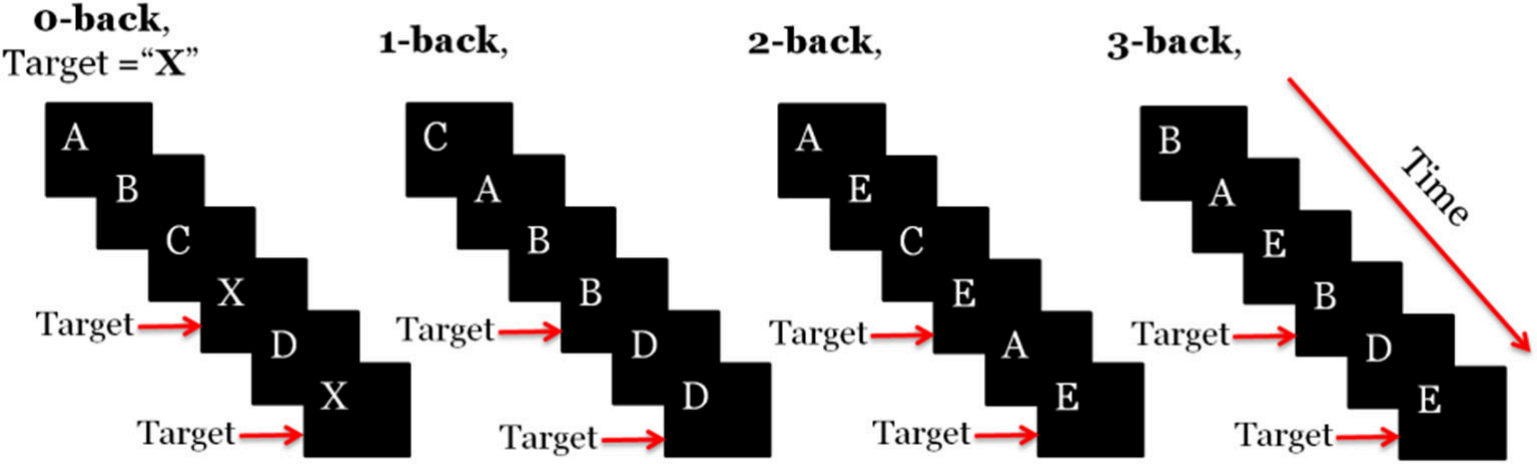
Schematic illustration of the letter *n*-back task for *n* ϵ {0, 1, 2, 3}.

One of the most common WM paradigm for MWL assessment is the *n*-back task, which was first introduced by Kirchner (1958). In the letter *n*-back task, participants observe a sequence of single letters separated by a certain amount of time each; for each letter they decide whether it is a target, i.e., identical to the item that appeared *n* items back in the sequence. The value of *n* is kept constant during a segment of the experiment referred to as a session. As *n* increases the difficulty of the task becomes higher. In the literature usually 0-back task has been used as a control state. Figure 1 illustrates how letter *n*-back task works when *n* is 0, 1, 2, or 3. Depending on *n*, subject should find the target letter and interact with the user-interface.

**Figure 2:**
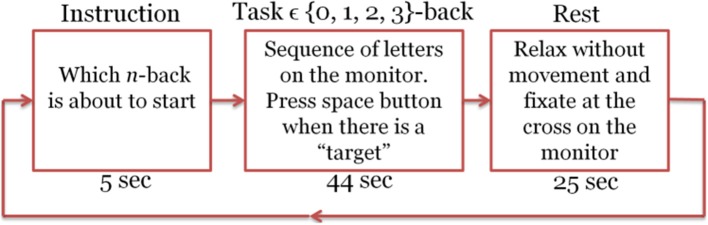
Experimental design for the letter *n*-back task. Each session includes the Instruction, task, and rest blocks.

In each experiment, we had a total number of 40 sessions. These sessions were presented in pseudorandom order, 10 sessions per *n*. Each session started with an instruction block that is displayed for 5 s on the screen and informed the subject about which type of the *n*-back tasks was about to start (instruction block). Then 22 randomly selected letters (out of 10 candidate pool of letters) appeared in sequence on the screen (task block). Each letter stayed on the screen for 500 ms and the subject had 1,500 ms to press the space button in case that the letter was a target according to the type of session. At the end of each session there was a 25 s resting block. During this block the subject remained relaxed and fixated at a cross on the screen to let the brain activation return to its baseline and get ready for the next *n*-back session (Herff et al., 2014). Figure 2 shows one sample session. Total recording time was 50 min. The program for implementation of this task was written using Presentation software (Neurobehavioral Systems, Inc.). All the information about the appearance time of each letter, session type, subject's response time, and also whether the presented letter was a target or not was recorded by this software and stored as a text file for later processing. The objective performance of the subjects within each session was computed from this information. Subjects who had too low accuracy (<90% in the 0- or <80% in the 1-back) were deemed insufficiently focused on the task. Performance level was measured by computing the accuracy defined as the fraction of correct responses. We considered a missed target as an incorrect response.

### Data acquisition and preprocessing

**Figure 3:**
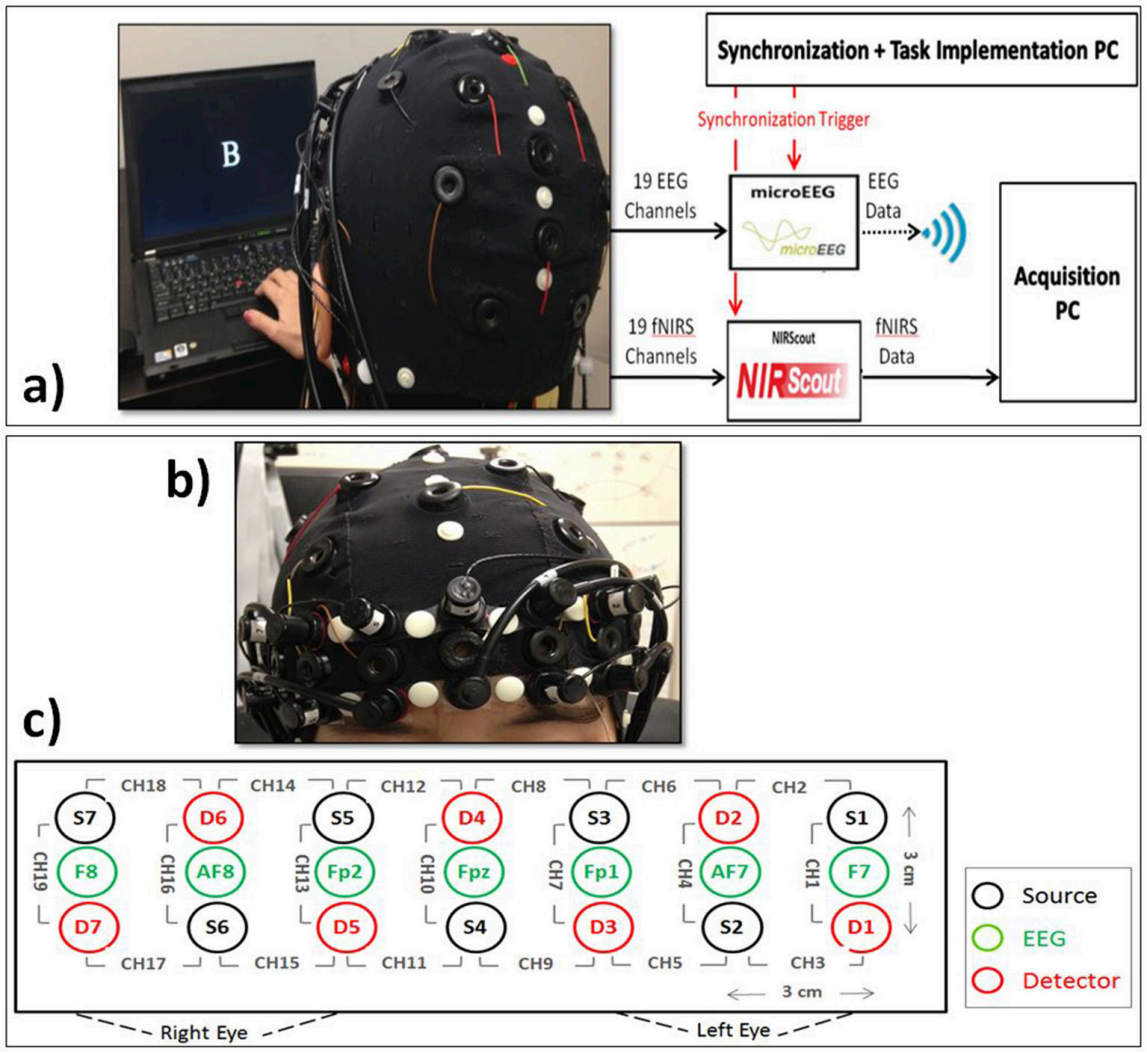
**(a)** EEG+fNIRS recording setup. Subject interaction with the computer, synchronization of EEG and fNIRS signal, recording of EEG and fNIRS signals, and data transmission to the acquisition platform. **(b)** Coronal view of the subject showing the close view of the placement fNIRS optodes and EEG electrodes. **(c)** Topographical view of fNIRS sources (S_i_, black) and detectors (D_i_, red) and EEG electrodes (green). Each pair of source and detector separated by 3 cm creates a channel (CH_i_). We used the signals from F7, Fpz, and F8.

A quantitative meta-analysis has found the cortical regions that were activated robustly during letter *n*-back task (Broadman Areas 6, 7, 8, 9, 10, 32, 40, 45, 46, 47, and supplementary motor area; Owen et al., 2005). We used this information together with the results of previous EEG studies to choose the optimum locations for our 19 EEG electrodes (F7, F8, F3, F4, Fz, Fc1, Fc2, T3, T4, C3, C4, Cp1, Cp2, P3, P4, Pz, Poz, O1, O2). We used Fpz as the ground and Cz as the reference electrode. In the literature, several different reference electrode positioning is indicated, which have their own set of strengths and weaknesses. Among them, linked ears and vertex (Cz) are the most common. Cz reference is advantageous when it is located in the middle among active electrodes, however, for close points, it may result in poor resolution (Teplan, 2002). Based on the previous studies, central brain region in not majorly involved during the performance of a WM task compared to the frontal and parietal lobes and choosing Cz as the reference may be more appropriate rather than any other electrode in the 10–20 system. microEEG (a portable device made by Bio-Signal Group Inc., Brooklyn, New York) was used to sample EEG at 250 Hz (Figure 3a). Electrode impedances were kept below 10 kΩ. A 128-channel electrode cap with Ag/AgCl electrodes (EasyCap, Germany) was used to physically stabilize the sensors and provide uniform scalp coverage. We located the fNIRS optodes on the subject's forehead to fully cover the PFC, which plays a significant role in WM (Fitzgibbon et al., 2013). Seven sources and seven detectors were located on the forehead resulting in 19 optical channels, each consisting of a source–detector (S–D) pair separated by a distance of 3 cm. The 19 optical channels used in this study are shown in Figure 3c. The S–D placement starts from the left hemisphere and ends on the right hemisphere. S4 and D4 are located at the center of forehead, where D4 is located at the AFz location and channel 10 is located at the Fpz location according to the standard international 10–20 system (Figures 3b,c). We used our triplet-holders (Keles et al., 2014a) on the forehead to keep each EEG electrode in the middle of an S–D pair and fix the distances between the sensors. fNIRS signals were acquired at 8.93 Hz via NIRScout extended (NIRx Medical Technologies, New York) device, which was synchronized with the EEG data by means of common event triggers (Figure 3a). NIRScout is a dual wavelength continuous wave system. The EEG signal was band-pass filtered (0.5–80 Hz), and a 60 Hz notch filter was used to reduce the power line noise.

**Figure 4:**
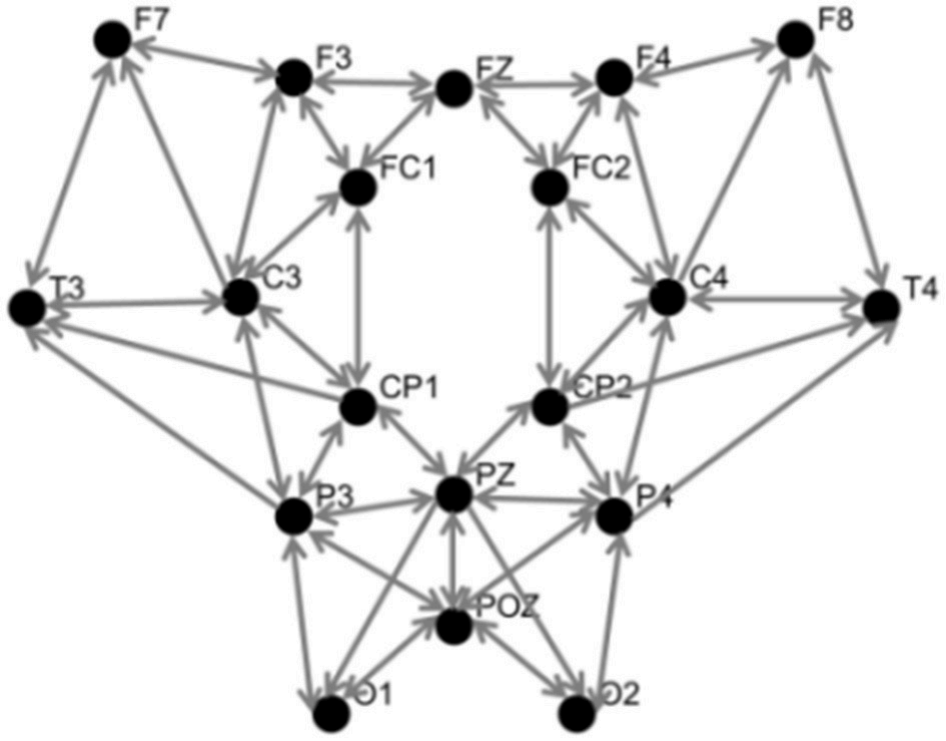
Topographic view of EEG electrodes showing neighborhood pattern for Laplacian spatial filtering. Inward arrows to each node indicate the corresponding neighbors used for spatial filtering.

**Figure 5:**
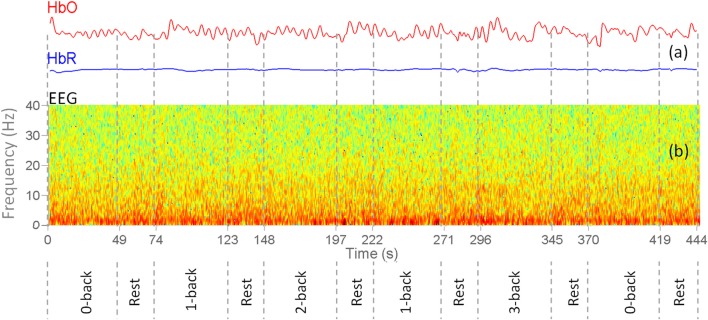
Sample preprocessed EEG+fNIRS data for one of the subjects. Vertical dashes separate different *n*-back task and rest blocks. **(a)** Concentration changes of oxy-hemoglobin (red curve) and deoxy-hemoglobin (blue) for channel 17. **(b)** EEG Time-frequency map of the channel O2.

The spatial Laplacian transform is generally effective in muscle artifact removal from EEG signal (Fitzgibbon et al., 2013). We subtracted the mean EEG voltage of the neighbor electrodes from each EEG signal. Figure 4 shows the configuration of neighbor electrodes for 19 EEG channels. Each detector in NIRScout device records the signal from each separate source in two different wavelengths (760 and 850 nm). Oxy- and deoxyhemoglobin concentration changes (HbO and HbR) were computed using the modified Beer-Lambert law (Sassaroli and Fantini, 2004) using standard values for the chromophore extinction coefficients and differential path-length factor (Keles et al., 2016). fNIRS might be contaminated with the movement, heart rate, and Mayer wave artifacts. In order to reduce these artifacts while retaining the maximum possible amount of information, a band pass filter of 0.01–0.5 Hz was applied to fNIRS signals. After the preprocessing step, two subjects were excluded from the rest of analysis due to the poor quality of the signal and excessive noise. The processed signals were inspected visually for the presence of muscle and motion, eye movements, and other artifacts. The recordings that were contaminated in excess of 10% by artifact were excluded as a whole (Keles et al., 2016). In addition, one subject was excluded since he was not sufficiently focused in the experiment according to 0-back low accuracy cut-off. Figure 5 shows a segment of preprocessed data for one of the subjects. The figure indicates the temporal variations in the fNIRS signals and the EEG frequency bands, which are utilized in feature extraction. First and second rows are HbO and HbR of fNIRS channel 17, respectively. Third row is the EEG time-frequency map for channel O2.

**Figure 6:**
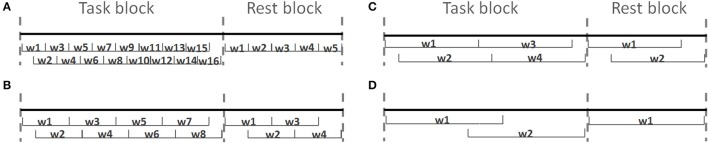
Four different epoch styles based on length of windows. The task and rest blocks are divided into **(A)** 5, **(B)** 10, **(C)** 20, and **(D)** 25 s windows (w_i_).

After preprocessing, each task block ({0, 1, 2, 3}-back) and rest block was divided into 5, 10, 20, or 25 s epochs in order to assess the effect of window size on classification results. Figure 6 shows four different epoch type with window size from 5 to 25 s. In most of the cases there is an overlap between adjacent epochs (half size of epoch's length). This overlap was considered in order to capture the unique temporal response for each individual, as there could be inter-subject variability in the time required for the hemodynamic response to peak, and/or in the number of peaks (Power et al., 2012). In addition, during the classification phase, an imbalance in the number of features within each class biases the training procedure in favor of the class with a higher number of training features (He and Garcia, 2009). In our experiment design we have 40 rest blocks and 10 blocks from each *n*-back task type. From each task block, 16, 8, 4, and 2 features were extracted when we changed the size of the window from 5 to 25 s, respectively. From each rest block 5, 4, 2, and 1 features were extracted when we changed the size of the window from 5 to 25 s, respectively.

### Feature extraction

We extracted from each window three main categories of features for all 19 EEG electrodes and 19 fNIRS channels: EEG (uni-modal), fNIRS (uni-modal), and EEG+fNIRS (multi-modal or Hybrid).

EEG-based features were computed from the frequency band power (PSD), phase locking value (PLV), phase-amplitude coupling (PAC), and the asymmetry of frequency band power between right and left hemispheres (Asym_PSD). Initially, the spectrogram was calculated using short-time Fourier transform method with windows of 1 s and half window size overlapping and frequency resolution of 1 Hz. The power was calculated in eight frequency bands each with a width of 4 Hz in the range 0 to 32 Hz. The ranges are referred to by their conventional labels: delta (0–4 Hz), theta (4–8), alpha (8–12), followed by five intervals ranging from low beta (12–16) to high beta (28–32). We also used the labels f1, f2,…, f8 for these frequency bands. EEG frequency band power for each epoch was extracted by integration of the corresponding power over each frequency band. We imposed the 32 Hz cutoff since higher frequencies in scalp EEG are generally not considered informative about cortical activity (Goncharova et al., 2003; Muthukumaraswamy, 2013). PLV is a measure of phase synchrony between two distinct neuronal populations, which is computed between two selected EEG electrodes as an estimate the inter-area synchrony (Vinck et al., 2011). PLV was estimated between electrode pairs that were selected to assess three different types of synchrony: intra-hemispheric (F3-P3, F4-P4, Fc1-Cp1, Fc2-Cp2, Fz-Poz), symmetric inter-hemispheric (F7-F8, F3-F4, Fc1-Fc2, C3-C4, T3-T4, Cp1-Cp2, P3-P4, O1-O2), and asymmetric inter-hemispheric (F3-P4, F4-P3, Fc1-Cp2, Fc2-Cp1). PLV was computed for four band of interest ([3–5], [9–11], [19–21], [39–41] Hz). PAC measures coupling between the phase of a low frequency (here [4–7], [9–13] Hz) oscillations and the amplitude of a high frequency ([15–35], [30–60] Hz) oscillation (Radwan et al., 2016). It provides an estimate of local, multi-frequency organization of neuronal activity (Dvorak and Fenton, 2014). We chose 8 EEG channel pairs between right and left hemispheres (F8-F7, F4-F3, Fc2-Fc1, T4-T3, C4-C3, Cp2-Cp1, P4-P3, and O2-O1) for Asym_PSD feature.

fNIRS features were based on HbO and HbR amplitude (HbO/R Amp.), slope of HbO and HbR (HbO/R slope), standard deviation of HbO and HbR (HbO/R Std.), skewness of HbO and HbR (HbO/R Skew.), and kurtosis of HbO and HbR (HbO/R Kurt.). The statistics of HbO and HbR are commonly used as features in fNIRS studies of MWL and BMIs (Naseer and Hong, 2015; Naseer et al., 2016a,b). Our inspection of the fNIRS data revealed patterns of correlation between HbO and HbR that were time and area dependent. Hence, we also included the zero-lagged correlation between HbO and HbR (HbO-HbR Corr.) as an additional feature. Hybrid features were based on EEG and fNIRS features in addition to specifically Hybrid quantities that depend simultaneously on both systems.

We chose to focus on a straightforward quantity, which can be easily calculated within the time windows of interest: the zero-lagged correlation between the Hb (HbO or HbR) amplitude and the EEG frequency band power (in eight separate bands described above). These neurovascular features based on HbO and HbR were denoted NVO (oxygenated neurovascular coupling) and NVR (deoxygenated neurovascular coupling), respectively. To calculate NVO/R for the left hemisphere, the correlation between each fNIRS channel (CH1 to CH9) and each frequency band of F7 EEG channel was calculated (band-passed filter within the specific frequency range). For the right hemisphere, the correlation between each fNIRS channel (CH11–CH19) and each frequency band of F8 EEG channel was calculated. For the fNIRS channel 10, which is located at the center we used the average of F7 and F8 channels to find NVO and NVR. This resulted in 152 (19 × 8) NVO and 152 NVR features from each window. Each set of features extracted from one subject's data were dc-shifted and scaled in order to have a mean value of zero and standard deviation of one.

### Classification and validation

Following feature extraction, we implemented SVM classification and *k*-fold cross-validation with *k* = 10. SVM is a non-parametric supervised classification method, which already showed promising results in the medical diagnostics, optical character recognition, electric load forecasting, and other fields. SVM can be a useful tool in the case of non-regularity in the data, for example when the data are not regularly distributed or have an unknown distribution (Auria and Moro, 2008). Linear SVM constructs an optimal hyperplane creating a decision surface maximizing the margin of separation between the closest data points belonging to different classes (Aghajani et al., 2013). The observations were randomly partitioned into *k* groups of approximately the same size. One group was selected as the testing and the rest as the training data. Principal component analysis (PCA) was applied to the training set. We applied PCA separately on each feature subgroup (11 subgroups, with the subgroups divided further by frequency bands as described in our feature extraction methods). For example, the EEG alpha frequency band power (8–12 Hz) consisted of the time series from 19 EEG channels. After PCA, these signals yielded 19 principal components (PC) and their associated time series as the new set of features. A similar PCA was applied to each feature subgroup. The resulting PCs contained a set of weights (for the EEG channels), which could be interpreted as an activation map. PCA therefore allowed us to interpret the topographic distribution of activation associated with every feature. PCA also yielded an eigenvalue corresponding to the variance of that feature. The eigenvalues typically decrease sharply, the sum of the first few accounting for almost all of the total energy of the 19 PCs. However, the most energetic PCs are not necessarily the most informative, as shown in Results (Table 1). In order to estimate the features' discriminating ability, we used the Pearson correlation coefficient method (Mwangi et al., 2014). A reference time series was constructed by labeling each window by a distinct integer that represented the rest or the task difficulty level ({0 (rest), 1 (0-back), 2 (1-back), 3 (2-back), 4 (3-back)}). We used *R*^2^, the square of the Pearson correlation between the time series and the reference signal, to rank the set of features. The testing data were projected into the PC space that was obtained from the training data and the testing features were ranked by using the same method. In part of our analysis, we have chosen to reduce the number of features of the systems (EEG, fNIRS, and Hybrid) by truncating all systems at the same fixed size, eliminating the lowest ranked features. The labeled training examples were fed into a binary linear SVM. Each training example contained a vector of feature values in a given window and its label that denoted one of the two classes of interest. The SVM constructed an optimal hyper-plane creating a decision surface maximizing the margin of separation between the closest data points belonging to different classes (Aghajani et al., 2013). In this study there were 10 possible pairs of binary classifications corresponding to our five distinct classes. In order to investigate the ability to discriminate WM loading against a baseline, we have chosen the pairs {1-back v rest}, {2-back v rest}, {3-back v rest}, {1-back v 0-back}, {2-back v 0-back}, and {3-back v 0-back} and performed binary classifications on them. We also investigated the ability to discriminate between degrees of MWL by using a multi-class scheme. For this purpose we utilized the error-correcting output code multiclass model (ECOC), which employs a set of binary classifiers. We adopted an all-pairs ECOC model to train a binary classifier on the pairs of classes in the training data and, for each new instance in the testing data, assigned the label that minimizes the aggregate Hamming loss from the predictions of all binary classifiers (Dietterich and Bakiri, 1995). In comparison to its alternatives, this approach has been shown to enhance accuracy while maintaining a low run-time complexity (Fürnkranz, 2002). We investigated four groups of multi-class sets that contained narrow gradations of MWL: {3-back v 2-back v 1-back}, {3-backv 2-back v 1-back v 0-back}, {3-back v 2-back v 1-back v rest}, and {3-back v 2-back v 1-back v 0-back v rest}. The accuracy was computed as the fraction of labels in the testing data that were correctly identified by the SVM. Finally, the cross-validation was repeated *k* times with each group of observations being used exactly once as the testing data. The overall accuracy was calculated as the mean of the repetitions. In addition to overall accuracy, confusion matrices yield a very detailed overview of a classifier's performance. Usually, the confusion matrix is further summarized by some proportions extracted from the confusion matrix. The main metrics are (a) sensitivity of class A (Sens._A_) which describes how well the classifier recognize observations of class A, (b) specificity of class A (Spec._A_) which describes how well the classifier recognizes that an observation does not belong to class A, (c) positive predictive value of class A (PPV_A_) tells us given the prediction is class A, what is the probability that the observation truly belongs to class A?, (d) negative predictive value of class A (NPV_A_) tells us given a prediction does not belong to class A, what is the probability that the sample truly does not belong to class A (Beleites et al., 2013)? We pooled all the *k* confusion matrices of the *k*-fold cross validation to calculate Sens._A_, Spec._A_, PPV_A_, and NPV_A_. For all the calculations described in this paper we used Matlab v.8.6.0.267246 (R2015b) (The MathWorks, Inc., Natick, Massachusetts, United States).

**Table 1:** The top *R*^2^ ranked features for three representative subjects for the binary rest v 3-back classification.

	**Subject 1**				**Subject 2**				**Subject 3**			
**Rank**	***R*^2^**	**Descr**.	**Freq. (Hz)**	**PC or Chan. Pair**	***R*^2^**	**Descr**.	**Freq. (Hz)**	**PC or Chan. Pair**	***R*^2^**	**Descr**.	**Freq. (Hz)**	**PC or Chan. Pair**
1	0.37	PSD	4–8	PC3	0.26	PSD	8–12	PC1	0.19	NVR	28–32	PC2
2	0.30	PSD	12–16	PC5	0.23	PSD	4–8	PC1	0.18	NVR	12–16	PC2
3	0.26	PSD	8–12	PC1	0.21	PSD	0–4	PC1	0.18	NVR	24–28	PC2
4	0.18	PLV	3–5	O1-O2	0.18	PSD	12–16	PC1	0.18	NVR	20–24	PC2
5	0.17	NVR	20–24	PC4	0.16	HbO	–	PC4	0.18	NVR	16–20	PC2
6	0.16	NVR	8–12	PC4	0.13	COR	–	PC2	0.18	HbR	–	PC2
7	0.16	NVR	16–20	PC4	0.13	HbR	–	PC1	0.17	NVR	4–8	PC2
8	0.16	NVR	12–16	PC4	0.12	NVR	24–28	PC1	0.16	PLV	9–11	Fz-Poz
9	0.16	NVR	24–28	PC4	0.11	NVR	12–16	PC1	0.15	PSD	12–16	PC3
10	0.15	NVR	28–32	PC4	0.11	NVR	16–20	PC1	0.14	PSD	8–12	PC4

*Columns indicate the feature description (Descr.), corresponding frequency range if applicable, and PC index in order of descending energy (or the channel pair for PLV). The HbO-HbR corr. has been abbreviated as COR*.

## Results

**Figure 7:**
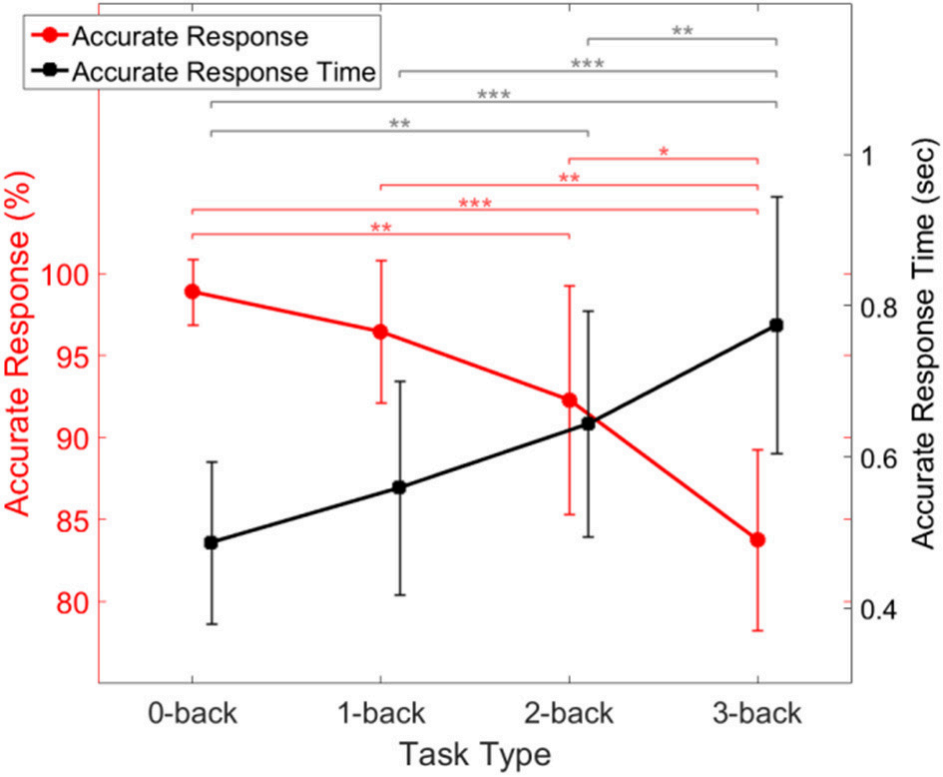
Behavioral performance of the subjects during task conditions of increasing difficulty, showing response accuracy (red) and response time (black). Error bars indicate the standard deviation of inter-subject variability. Asterisks indicate statistical significance derived from a two-way ANOVA comparison of each two response accuracy (red) or response time (black) (^*^p < 0.05, ^**^p < 0.001, ^***^p < 0.0001).

We initially investigated the relationship between the subjects' performance and task difficulty, in order to insure that it was consistent with expectations. Figure 7 shows the accuracy and response time of all subjects with error bars showing the standard deviation of inter-subject variability. The figure shows that the fraction of accurate responses decreased with increasing task difficulty. There was little or no accuracy decrement between 0- and 1-back tasks, as expected (Jonides et al., 1997). Furthermore, the time it took subjects to produce a correct response increased (and eventually more than doubled) with task difficulty (Figure 7).

**Figure 8:**
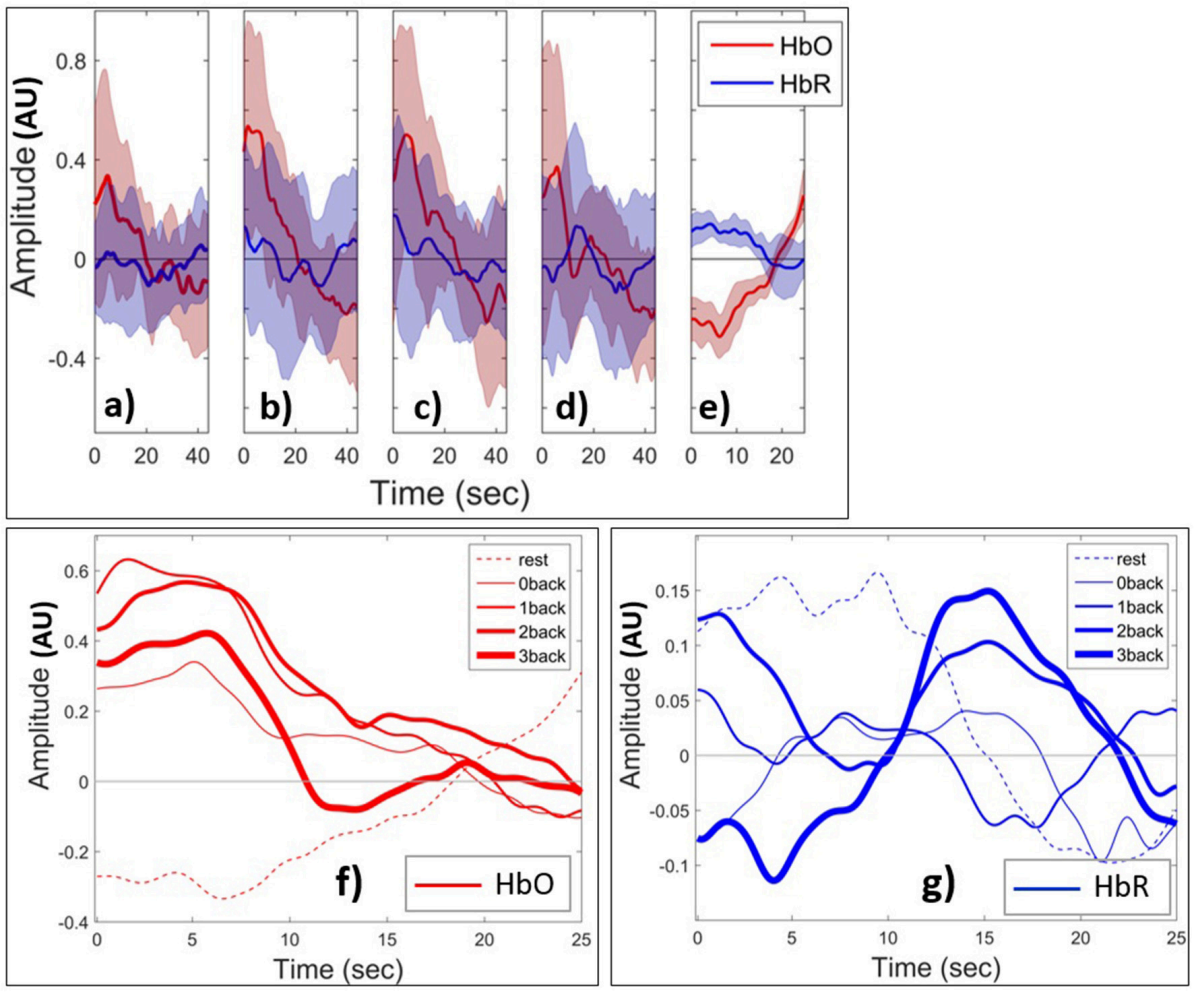
Grand block average of normalized HbO (red) and HbR (blue) during **(a)** 0-back, **(b)** 1-back, **(c)** 2-back, **(d)** 3-back, **(e)** rest. The thick curves show the average over all channels and subjects. The shaded area indicates the standard deviation of inter-subject variability. Grand block average of HbO **(f)** and HbR **(g)** for rest (dashed curves) and task (solid). Increasing thickness of solid curves corresponds to increasing task difficulty from 0- to 3-back. AU means arbitrary units.

We next examined the HbO and HbR patterns during changes in mental load. Figures 8 (a–e) shows the grand block average of HbO (red) and HbR (blue) amplitude. The shaded area shows the standard deviation of the inter-subject variability. In this paper, the term grand block averaging denotes the average over the blocks of the same class and over all channels and subjects. Following neural activation, local blood flow and volume typically increase on a time scale of seconds and, at the beginning of the task, there is a localized rise in oxygenation in PFC (Huppert et al., 2006), which creates the positive peak of HbO. After a few seconds due to the metabolic consumption of oxygen the oxyhemoglobin concentration decreases leading to a negative HbO amplitude. During the rest state which comes after the task block, the oxyhemoglobin concentration starts to increase and HbO returns to baseline. Toward the end of rest window there is an apparent task anticipating rise in HbO. The range of changes of HbO is obviously higher than those of HbR during the task periods. From 0- to 2-back the positive peak of HbO increases and then decreases from 2- to 3-back. HbO and HbR have the opposite sign and are hence negatively short time correlated in the rest state. However, this appears to change during task in ways depending on the value of *n*. The range of HbO changes increases with *n* although they appear to slightly decrease as *n* changes from 2 to 3. In (Figures 8f,g) we show the grand block average of all tasks v rest state for one specific fNIRS channel (channel 10, which is located at Fpz, near the center of the forehead). In this figure the curves corresponding to rest and task with all values of *n* have been shown in one plot to make the comparison easier. And we just show the first 25 s of the *n*-back task block (out of the full 44 s). The shaded areas for standard deviation are omitted for clarity. Figure 8f shows that the peak amplitude of HbO is positive for task performance and negative for rest. In addition, it decreases with increasing load for *n* > 0. The area under curve clearly discriminates between rest and task since it is negative during rest and positive for all other *n*-back tasks. By contrast, the peaks of the amplitude of grand block average of HbR (Figure 8g) that occur after the 10 s have a positive correlation with the level of mental load. These patterns are not observed in the case of the 0-back task since it is related to perception only and is less involved with WM system. We also examined the time course of selected features that were extracted from the signals.

**Figure 9:**
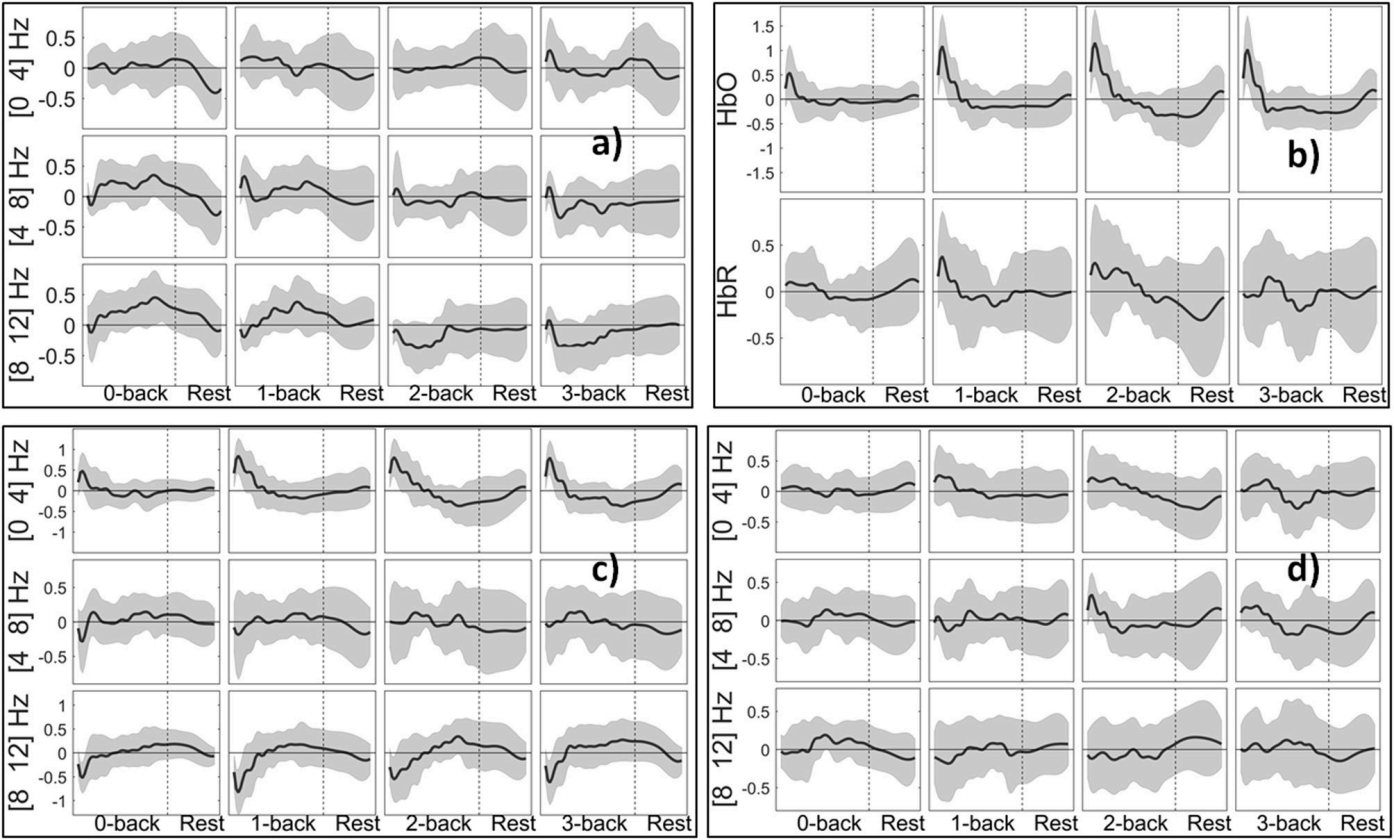
Grand block average of normalized features from 5 s windows: **(a)** PSD (delta, theta, alpha bands) of channel O2. **(b)** HbO/R Amp. for channel 10. **(c)** NVO (delta, theta, alpha bands) for channel 10. **(d)** NVR features (delta, theta, alpha bands) for channel 10. Shaded areas indicate the standard deviation of inter-subject variability.

Figure 9 shows the PSD extracted from EEG, HbO/R Amp. from fNIRS, and NVO/R features from EEG+fNIRS change in relation to the degree of WM load. We use the term session to denote a task block and the following rest block. For each cognitive state, we then have 10 sessions per subject. In the case of 5 s windowing, for each feature, we have 21 values in each session (16 from task block and 5 from rest block). The curves in Figure 9 were computed by first applying a simple triangular moving average filter covering three samples at each step, and then a cubic spline interpolation. The figure shows that the theta and alpha bands of EEG are positive during 0- and 1-back, although they become negative for 2- and 3-back tasks. The positive peak of HbO increases from 0- to 2-back and has a slightly lower peak for 3-back compared to 2-back. The figure also shows that the Hybrid features (such as NVO in the delta range) generally resemble the corresponding uni-modal features (such as HbO and PSD in the delta range) however they were dominated by neither, suggesting that the Hybrid feature contained additional information.

Table 1 shows the top 10 highest ranked features (based on *R*^2^) for three subjects obtained during the 3-back v rest training set. The features are characterized by the description (e.g., PSD, PLV, HbO, as described in Section Methods) and the particular frequency band, where applicable. The frequency band is applicable only to the EEG and neurovascular features. The table also indicates the order of the feature according to the magnitude of its eigenvalue [ordered from the most energetic (1) to the least (19)]. Since PCA was not used in the case of PLV, the channel label is given instead of the PC order. For example, the highest ranked feature for subject one was the third most energetic PC from the EEG frequency band power in the theta range (4–8 Hz). For subject 3, the highest ranked feature was the second most energetic PC from the neurovascular feature based on the correlation between HbR and the EEG frequency band power in the high beta range (28–32 Hz). The table illustrates that the types of features in the top ranked group may vary among subjects and that high discriminating ability of a feature does not imply high energy in the sense of the PCA.

**Figure 10:**
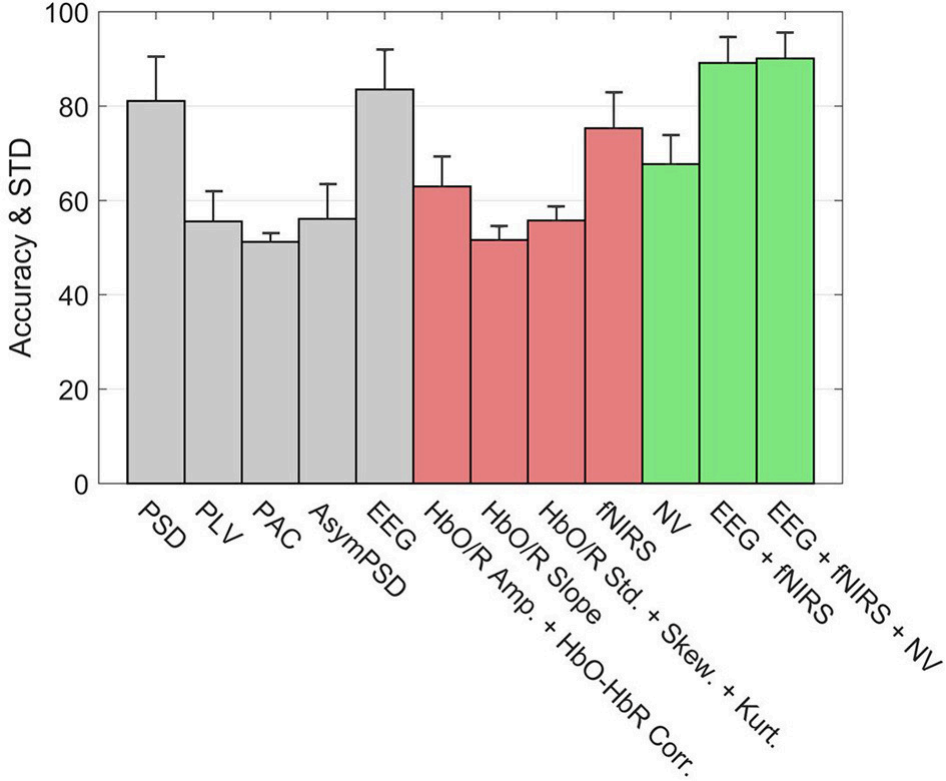
Accuracy of types of features in classifying rest v 3-back with 5 s feature windows. The error bars indicate the standard deviation of inter-subject variability. The union of neurovascular features is abbreviated as NV. Features are extracted from different systems: EEG (gray bars), fNIRS (red bars), and Hybrid (green bars).

Figure 10 shows the classification accuracies of various subsystems as well as the Hybrid system for the 3-back v rest using 5 s windows. The error bars represent the standard deviation of inter-subject variability. Within the EEG group (gray bars), the leftmost bar is the accuracy of a system based only the PSD features. On its immediate right is the accuracy of the subsystem based only on PLV features, and similarly for PAC and other feature types. The rightmost bar in the EEG group shows the accuracy of the full EEG system which includes all feature types based on EEG signals. Clearly the PSD is the primary contributor to the discriminating ability of the EEG, however, the accuracy appears to be slightly enhanced by including the other types of features. Among the fNIRS systems (red) the leftmost bar indicates that Hb amplitudes together with the HbO-HbR correlation is the primary contributor to the accuracy of detection. Unlike the EEG system, the other feature types such as slope and higher order statistics significantly enhance the accuracy of the fNIRS system. The overall accuracy of the fNIRS system is lower than the overall accuracy of the EEG system. The accuracy based only on the neurovascular features is indicated by the leftmost bar in the Hybrid group (green). The middle bar in the Hybrid group represents the pooling of all features from the EEG and fNIRS systems. Finally the inclusion of the neurovascular features in the Hybrid system (rightmost green bar) appears to slightly enhance the accuracy.

**Figure 11:**
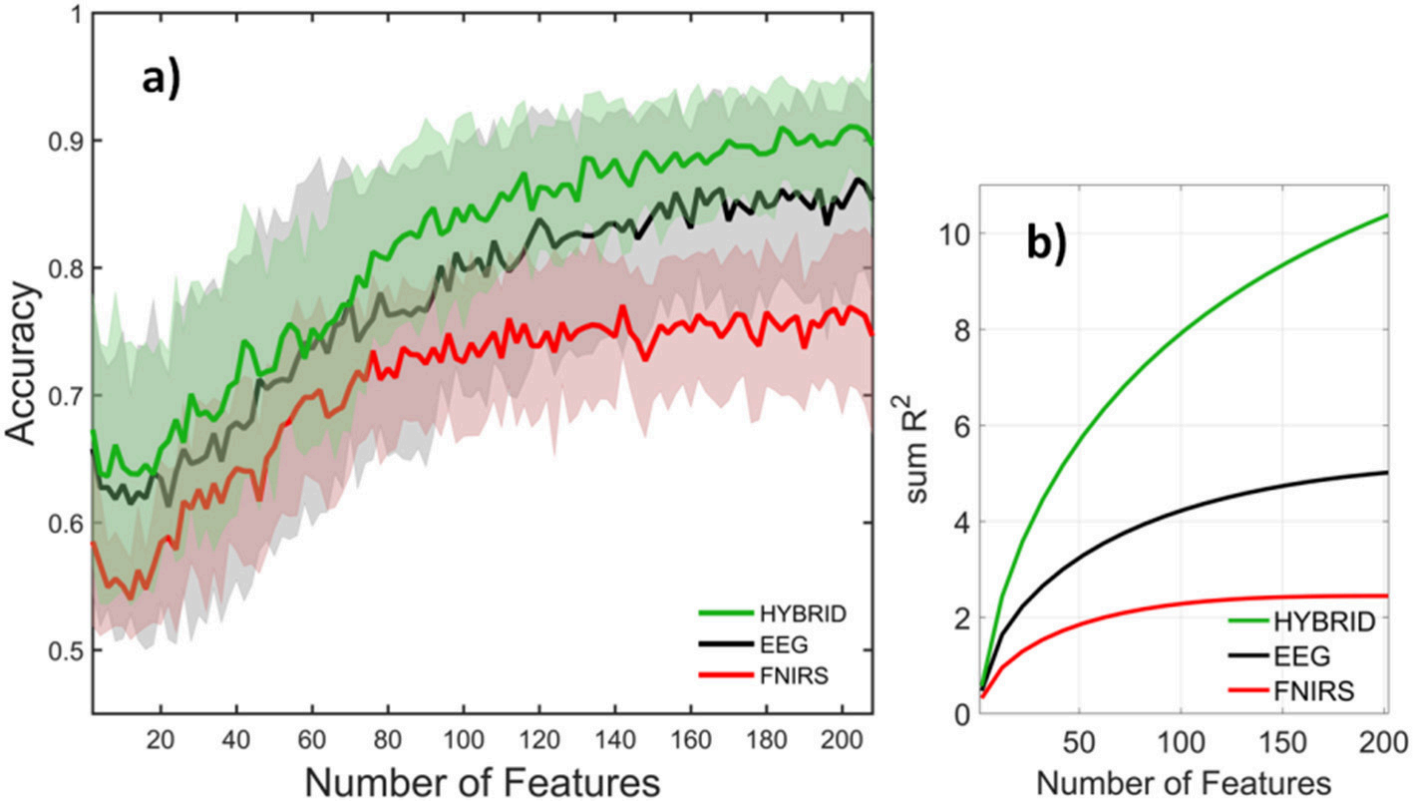
**(a)** Accuracy and **(b)** cumulative sum of *R*^2^ for EEG (black), fNIRS (red), and Hybrid (green) systems as a function of system size. Mean and standard deviation over subjects are indicated by the solid curves and shaded areas, respectively. The classification task was rest v 3-back and feature window size was 5 s.

Figure 10 compared the accuracies of various systems with each system containing the full set of features that belonged to it. The number of features in the full set was different for each system, for example the EEG, fNIRS, and Hybrid systems contained 360, 209, and 873 features, respectively. This may raise the question of to what extent these systems' accuracies were influenced by the number of features they contained, rather than by the information content per feature. In order to examine this topic, we computed the systems' accuracies after they had been truncated to contain the same number of features. This was done by selecting the top ranking group after the features had been sorted in order of descending values of *R*^2^. The goal was to perform a comparison on an equal footing by truncating each system in the same way. The calculation was repeated by varying the number of features from two to the available maximum. Figure 11a indicates that the fNIRS system had the lowest accuracy over the entire range of the number of features. The EEG system performed better, while the Hybrid accuracy was consistently superior to either system, similar to the results shown in Figure 10. Figure 11b shows the cumulative sums of *R*^2^ index v number of features for three systems which qualitatively agree with Figure 11a. The calculations are for the 3-back v rest using 5 s windows and they qualitatively agree with the results (not shown) of binary classifications of other pairs of classes and window sizes. The shaded areas indicate the standard deviation of inter-subject variability.

Figure 10 provided the results for only one type of binary classification (3-back v rest) and the variability over subjects as a standard deviation. However, it is highly instructive to examine the result for each subject as well as for every binary and multi-class problem that was described previously in our Methods. Tables 2, 3 break down the accuracy of classification for each subject (S1, S2,…, S14), system type (EEG, fNIRS, Hybrid), and the type of classification problem. The mean as well as the minimum and maximum of the values for the subject population are provided as three separate columns on the left. The height of the rightmost bars within the EEG (gray), fNIRS (red), and Hybrid (green) groups in Figure 10 correspond in Table 2 to the accuracy percentages 83.5, 75.3, and 90.1 shown under the column “Mean” and the row “3-back v rest.” In the columns for individual subjects, Table 2 shows the mean accuracy and the standard deviation from the trials in the 10-fold cross validation.

**Table 2:** Binary classification accuracy for all subjects included in the study (S1 to S14) for 10-fold cross validation.

		**Min**	**Max**	**Mean**	**S1**	**S2**	**S3**	**S4**	**S5**	**S6**	**S7**	**S8**	**S9**	**S10**	**S11**	**S12**	**S13**	**S14**
1back v rest	EEG	65.9	92.5	78.9	81.3 ± 14	89.1 ± 13	73.1 ± 17	65.9 ± 13	84.4 ± 13	92.5 ± 4	90.6 ± 6	67.2 ± 15	74.1 ± 17	72.5 ± 17	91.3 ± 9	75.0 ± 14	80.0 ± 19	68.1 ± 13
	fNIRS	67.5	89.4	75.3	71.9 ± 13	70.0 ± 14	68.8 ± 11	69.7 ± 13	69.7 ± 11	82.2 ± 14	86.9 ± 5	80.6 ± 8	67.5 ± 14	69.1 ± 15	89.4 ± 10	83.1 ± 7	70.6 ± 14	75.0 ± 12
	Hybrid	76.5	97.2	87.2	89.1 ± 12	79.7 ± 18	76.6 ± 15	86.9 ± 12	76.6 ± 17	95.6 ± 4	90.6 ± 14	86.9 ± 9	86.9 ± 10	77.8 ± 16	97.2 ± 4	91.9 ± 8	93.8 ± 4	90.9 ± 8
2back v rest	EEG	69.4	95.3	82.9	90.6 ± 9	87.5 ± 9	86.3 ± 9	75.3 ± 10	87.5 ± 8	92.5 ± 11	83.4 ± 9	69.4 ± 15	72.2 ± 12	75.6 ± 16	95.3 ± 4	79.7 ± 12	92.2 ± 6	73.1 ± 15
	fNIRS	70.0	84.7	77.6	79.1 ± 15	70.0 ± 15	72.2 ± 11	71.6 ± 15	80.3 ± 13	84.7 ± 10	79.4 ± 7	78.1 ± 9	73.8 ± 16	78.8 ± 10	84.7 ± 11	74.1 ± 14	82.8 ± 5	77.2 ± 15
	Hybrid	71.2	97.2	89.3	91.6 ± 8	71.3 ± 17	92.5 ± 5	87.2 ± 12	92.2 ± 5	97.2 ± 3	87.2 ± 9	82.8 ± 12	84.7 ± 12	92.5 ± 5	93.4 ± 8	90.9 ± 4	92.2 ± 9	94.4 ± 5
3back v rest	EEG	67.2	96.6	83.5	90.9 ± 6	78.8 ± 17	82.2 ± 18	82.8 ± 12	82.2 ± 10	96.6 ± 6	87.2 ± 14	67.2 ± 12	88.8 ± 14	74.1 ± 15	94.4 ± 10	80.3 ± 24	89.7 ± 9	74.4 ± 19
	fNIRS	63.7	88.4	75.3	67.5 ± 16	68.4 ± 9	65.6 ± 13	63.7 ± 14	83.8 ± 5	82.5 ± 11	77.2 ± 11	78.1 ± 11	75.0 ± 13	82.2 ± 11	88.4 ± 12	78.8 ± 11	72.5 ± 11	70.6 ± 14
	Hybrid	82.2	96.6	90.1	95.6 ± 2	84.7 ± 17	89.4 ± 14	83.8 ± 17	92.8 ± 5	95.6 ± 5	82.5 ± 15	82.2 ± 14	93.4 ± 7	84.7 ± 15	96.6 ± 4	92.2 ± 4	94.7 ± 4	93.4 ± 6
1back v 0back	EEG	80.0	91.9	86.7	89.1 ± 5	91.9 ± 4	80.0 ± 8	87.5 ± 5	89.7 ± 4	84.7 ± 5	87.2 ± 6	83.4 ± 6	86.6 ± 6	81.6 ± 8	85.3 ± 5	90.3 ± 7	88.1 ± 5	88.8 ± 5
	fNIRS	67.8	82.8	74.0	68.8 ± 10	74.1 ± 12	73.4 ± 9	69.7 ± 10	70.9 ± 7	76.6 ± 7	74.1 ± 11	82.8 ± 6	70.0 ± 9	73.4 ± 11	77.8 ± 8	79.7 ± 5	67.8 ± 12	77.2 ± 8
	Hybrid	88.1	93.4	91.4	90.9 ± 5	91.6 ± 6	89.1 ± 5	91.3 ± 5	93.1 ± 4	92.2 ± 4	92.5 ± 4	93.4 ± 4	88.1 ± 7	88.1 ± 6	91.9 ± 5	93.1 ± 6	93.1 ± 6	91.9 ± 3
2back v 0back	EEG	85.0	94.7	91.2	89.4 ± 5	89.7 ± 5	94.7 ± 3	87.8 ± 5	93.1 ± 5	94.7 ± 5	92.2 ± 5	89.1 ± 5	91.9 ± 4	85.0 ± 5	93.8 ± 5	92.2 ± 4	93.8 ± 5	89.1 ± 3
	fNIRS	66.9	83.4	74.8	66.9 ± 8	72.8 ± 7	71.3 ± 5	69.7 ± 5	78.1 ± 11	77.2 ± 6	73.8 ± 8	75.3 ± 9	79.4 ± 6	72.8 ± 7	79.7 ± 5	83.4 ± 7	70.3 ± 10	77.2 ± 8
	Hybrid	91.6	96.6	93.8	91.9 ± 6	91.6 ± 4	93.8 ± 4	91.9 ± 6	94.7 ± 3	95.3 ± 5	92.8 ± 6	94.4 ± 4	93.8 ± 4	92.5 ± 4	96.6 ± 3	95.6 ± 4	94.7 ± 4	93.4 ± 5
3back v 0back	EEG	86.2	96.2	92.0	91.9 ± 6	86.3 ± 5	90.9 ± 4	90.3 ± 4	92.5 ± 3	95.3 ± 5	94.1 ± 6	88.4 ± 8	93.4 ± 4	86.3 ± 7	96.3 ± 6	94.4 ± 4	94.4 ± 4	93.1 ± 4
	fNIRS	66.6	77.5	71.6	66.9 ± 4	67.2 ± 8	77.5 ± 5	67.5 ± 8	74.1 ± 6	74.7 ± 7	71.9 ± 10	70.3 ± 6	74.7 ± 8	75.9 ± 6	73.8 ± 9	72.5 ± 9	68.8 ± 8	66.6 ± 9
	Hybrid	90.6	96.6	93.8	93.4 ± 5	90.6 ± 5	96.3 ± 2	92.2 ± 4	94.7 ± 3	95.9 ± 3	92.8 ± 5	91.3 ± 5	93.4 ± 5	91.9 ± 3	93.8 ± 5	95.0 ± 3	94.7 ± 4	96.6 ± 3

*For each one of the subjects, ANOVA showed a significant difference (p < 0.0001) for comparison of EEG v Hybrid results and fNIRS v Hybrid results*.

**Table 3:** Multi-class classification accuracy for all subjects included in the study (S1 to S14) for 10-fold cross validation.

		**Min**	**Max**	**Mean**	**S1**	**S2**	**S3**	**S4**	**S5**	**S6**	**S7**	**S8**	**S9**	**S10**	**S11**	**S12**	**S13**	**S14**
3back v 2back v 1back	EEG	72.9	85.4	80.8	76.5 ± 7	84.2 ± 5	74.2 ± 8	76.7 ± 7	82.3 ± 6	83.8 ± 6	85.4 ± 4	85.2 ± 4	83.5 ± 4	72.9 ± 6	83.5 ± 6	81.3 ± 7	82.5 ± 5	80.0 ± 8
	fNIRS	49.6	62.5	57.9	49.6 ± 8	61.3 ± 8	54.0 ± 10	56.3 ± 9	60.0 ± 6	62.5 ± 8	54.4 ± 8	58.8 ± 8	56.9 ± 6	55.0 ± 7	62.1 ± 8	60.2 ± 8	61.3 ± 10	59.0 ± 8
	Hybrid	82.5	90.2	87.3	82.5 ± 3	90.2 ± 4	85.4 ± 3	85.2 ± 5	90.0 ± 5	88.1 ± 3	88.7 ± 5	88.5 ± 4	85.4 ± 4	84.6 ± 5	90.2 ± 5	86.9 ± 5	87.3 ± 4	89.8 ± 5
3back v 2back v 1back v Rest	EEG	73.1	87.7	80.6	80.6 ± 5	85.6 ± 3	77.3 ± 6	76.7 ± 5	80.5 ± 5	87.7 ± 4	85.5 ± 5	74.5 ± 6	82.2 ± 4	73.1 ± 5	86.3 ± 4	77.5 ± 4	84.7 ± 4	76.3 ± 4
	fNIRS	55.8	69.5	62.3	58.4 ± 4	61.7 ± 3	57.5 ± 6	61.1 ± 6	64.7 ± 6	66.7 ± 5	55.8 ± 4	62.5 ± 4	61.3 ± 8	58.3 ± 4	69.5 ± 5	65.3 ± 7	63.7 ± 6	65.2 ± 4
	Hybrid	82.7	91.7	87.1	85.2 ± 5	88.4 ± 2	86.3 ± 3	85.0 ± 4	87.7 ± 4	89.1 ± 3	86.9 ± 4	82.7 ± 4	88.1 ± 2	84.1 ± 4	91.7 ± 3	86.6 ± 5	88.3 ± 4	89.7 ± 2
3back v 2back v 1back v 0back	EEG	70.3	83.9	79.2	77.5 ± 6	80.8 ± 4	72.3 ± 5	77.3 ± 6	79.7 ± 5	82.8 ± 2	83.4 ± 5	77.8 ± 3	80.2 ± 5	70.3 ± 5	82.5 ± 3	82.0 ± 6	83.9 ± 4	78.8 ± 4
	fNIRS	42.5	56.2	51.7	42.5 ± 5	51.2 ± 5	49.2 ± 7	48.4 ± 8	56.3 ± 6	53.3 ± 5	48.0 ± 6	55.8 ± 7	50.5 ± 10	49.8 ± 6	56.3 ± 7	55.5 ± 5	55.0 ± 7	52.3 ± 5
	Hybrid	81.4	88.9	84.9	81.7 ± 5	85.3 ± 4	82.8 ± 4	81.4 ± 4	87.5 ± 3	85.0 ± 5	83.9 ± 3	84.7 ± 2	84.8 ± 4	81.6 ± 7	88.9 ± 4	87.3 ± 4	86.1 ± 4	87.5 ± 3
3back v 2back v 1back v 0back v rest	EEG	69.6	85.0	78.0	79.0 ± 5	82.4 ± 5	73.0 ± 4	72.7 ± 3	77.4 ± 5	84.5 ± 2	82.3 ± 3	72.5 ± 4	79.9 ± 2	69.6 ± 5	85.0 ± 4	78.6 ± 3	83.8 ± 4	72.0 ± 4
	fNIRS	46.7	62.6	56.2	46.8 ± 7	55.8 ± 5	54.6 ± 7	53.0 ± 5	59.0 ± 5	62.6 ± 3	54.0 ± 3	57.4 ± 4	54.6 ± 6	52.5 ± 6	61.9 ± 3	59.9 ± 5	57.1 ± 5	58.4 ± 5
	Hybrid	81.1	92.1	85.4	85.5 ± 6	84.3 ± 3	83.0 ± 3	82.5 ± 3	86.0 ± 3	87.9 ± 3	85.1 ± 3	81.1 ± 2	84.2 ± 4	81.7 ± 5	92.1 ± 3	87.1 ± 3	86.5 ± 4	88.3 ± 4

*For each one of the subjects, ANOVA showed a significant difference (p < 0.0001) for comparison of EEG v Hybrid results and fNIRS v Hybrid results*.

Table 2 suggests that the mean accuracy of classifying task against a baseline increases with *n*, as expected. The accuracy of detecting 0-back v *n*-back appears to be slightly greater than that of detecting rest v *n*-back (*n* > 0). For example, 87.2% for 1-back v rest and 91.4% for 1-back v 0-back. Table 3 shows the results for multi-class classification. In this case, the accuracy tends to decline slightly as more classes are included in the classification problem. In all subjects and classification problems, the Hybrid system has the greatest accuracy without exception. We investigated whether the observed superiority of the Hybrid system was statistically significant. A two-way ANOVA was performed on every classification problem (a row of Table 2 or Table 3) by using as the two factors the type of system (EEG, fNIRS, Hybrid) and the subject. The analysis was repeated by taking the classification problems as, first, the binary types in Table 2 and, second, the multi-class types in Table 3. In all cases, the differences of accuracy among the subjects were not significant and there were no interactions between system type and subject, while the differences in accuracy between the Hybrid and the uni-modal system was significant with a *p* < 0.001.

Table 4 lists the sensitivity (Sens.), specificity (Spec.), positive predictive value (PPV), and negative predictive value (NPV) for each individual class within a classification case. For example for the case of {Rest v 3back}, each one of rest and 3-back classes would have a Sens., Spec., PPV, and NPV, respectively. In addition, this table summarizes all these metrics for EEG, fNIRS, and Hybrid systems in order to make it easier to compare between their capabilities.

**Table 4:** Sensitivity (Sens.), specificity (Spec.), positive predictive value (PPV), and negative predictive value (NPV) are listed in percentage (%) for all classification cases (binary and multi-class) and all systems (EEG, fNIRS, Hybrid).

		**Sens**.			**Spec**.			**PPV**			**NPV**		
		**EEG**	**fNIRS**	**Hybrid**	**EEG**	**fNIRS**	**Hybrid**	**EEG**	**fNIRS**	**Hybrid**	**EEG**	**fNIRS**	**Hybrid**
Rest v 1back	Rest	94.5	94.5	96.7	63.3	56.7	79.6	72.1	68.6	82.6	92.0	91.1	96.1
	1back	63.3	56.7	79.6	94.5	94.5	96.7	92.0	91.1	96.1	72.1	68.6	82.6
Rest v 2back	Rest	94.0	95.2	95.8	71.8	56.7	82.5	76.9	68.7	84.6	92.3	92.2	95.1
	2back	71.8	56.7	82.5	94.0	95.2	95.8	92.3	92.2	95.1	76.9	68.7	84.6
Rest v 3back	Rest	94.4	94.2	96.6	72.7	56.7	83.3	77.5	68.5	85.2	92.8	90.8	96.1
	3back	72.7	56.7	83.3	94.4	94.2	96.6	92.8	90.8	96.1	77.5	68.5	85.2
0back v 1back	0back	86.7	69.2	90.9	86.7	72.9	92.4	86.7	71.9	92.3	86.7	70.3	91.0
	1back	86.7	72.9	92.4	86.7	69.2	90.9	86.7	70.3	91.0	86.7	71.9	92.3
0back v 2back	0back	90.8	67.5	92.5	91.5	76.7	94.3	91.4	74.3	94.2	90.9	70.3	92.6
	2back	91.5	76.7	94.3	90.8	67.5	92.5	90.9	70.3	92.6	91.4	74.3	94.2
0back v 3back	0back	90.7	68.8	93.0	93.3	71.2	94.0	93.1	70.5	93.9	90.9	69.6	93.1
	3back	93.3	71.2	94.0	90.7	68.8	93.0	90.9	69.6	93.1	93.1	70.5	93.9
1back v 2back v 3back	1back	76.0	51.1	82.5	94.4	79.3	95.5	87.1	55.3	90.1	88.7	76.4	91.6
	2back	83.8	60.4	89.6	87.7	78.3	90.9	77.3	58.1	83.1	91.6	79.8	94.6
	3back	82.7	57.8	87.1	89.2	77.1	93.2	79.3	55.7	86.5	91.2	78.5	93.5
0back v 1back v 2back v 3back	0back	76.4	45.0	81.4	94.6	85.4	96.0	82.6	50.7	87.1	92.3	82.3	93.9
	1back	79.9	48.1	86.7	91.7	82.6	93.9	76.2	48.0	82.5	93.2	82.7	95.5
	2back	81.0	53.4	87.0	92.3	81.5	94.2	77.9	49.1	83.4	93.6	84.0	95.6
	3back	79.7	50.7	83.8	93.7	82.8	95.5	80.8	49.5	86.2	93.3	83.4	94.6
Rest v 1back v 2back v 3back	Rest	81.4	79.6	86.4	95.4	91.5	97.3	85.5	75.8	91.4	93.9	93.1	95.6
	1back	79.3	51.6	86.6	93.9	85.1	95.6	81.3	53.5	86.8	93.2	84.0	95.5
	2back	80.8	56.0	87.0	92.3	85.8	94.7	77.8	56.9	84.5	93.5	85.4	95.6
	3back	80.8	54.7	86.4	92.5	84.9	94.6	78.3	54.7	84.2	93.5	84.9	95.4
Rest v 0back v 1back v 2back v 3back	Rest	78.6	77.9	84.5	95.3	93.4	97.5	80.8	74.8	89.3	94.7	94.4	96.2
	0back	79.4	47.3	85.4	94.2	87.5	95.9	77.5	48.7	84.0	94.8	86.9	96.3
	1back	78.0	44.8	84.4	93.7	86.9	95.3	75.7	46.0	81.8	94.5	86.3	96.1
	2back	75.9	49.9	84.3	94.4	87.7	96.1	77.3	50.4	84.6	94.0	87.5	96.1
	3back	78.3	48.8	85.2	94.8	86.6	96.1	79.1	47.8	84.5	94.6	87.1	96.3

**Figure 12:**
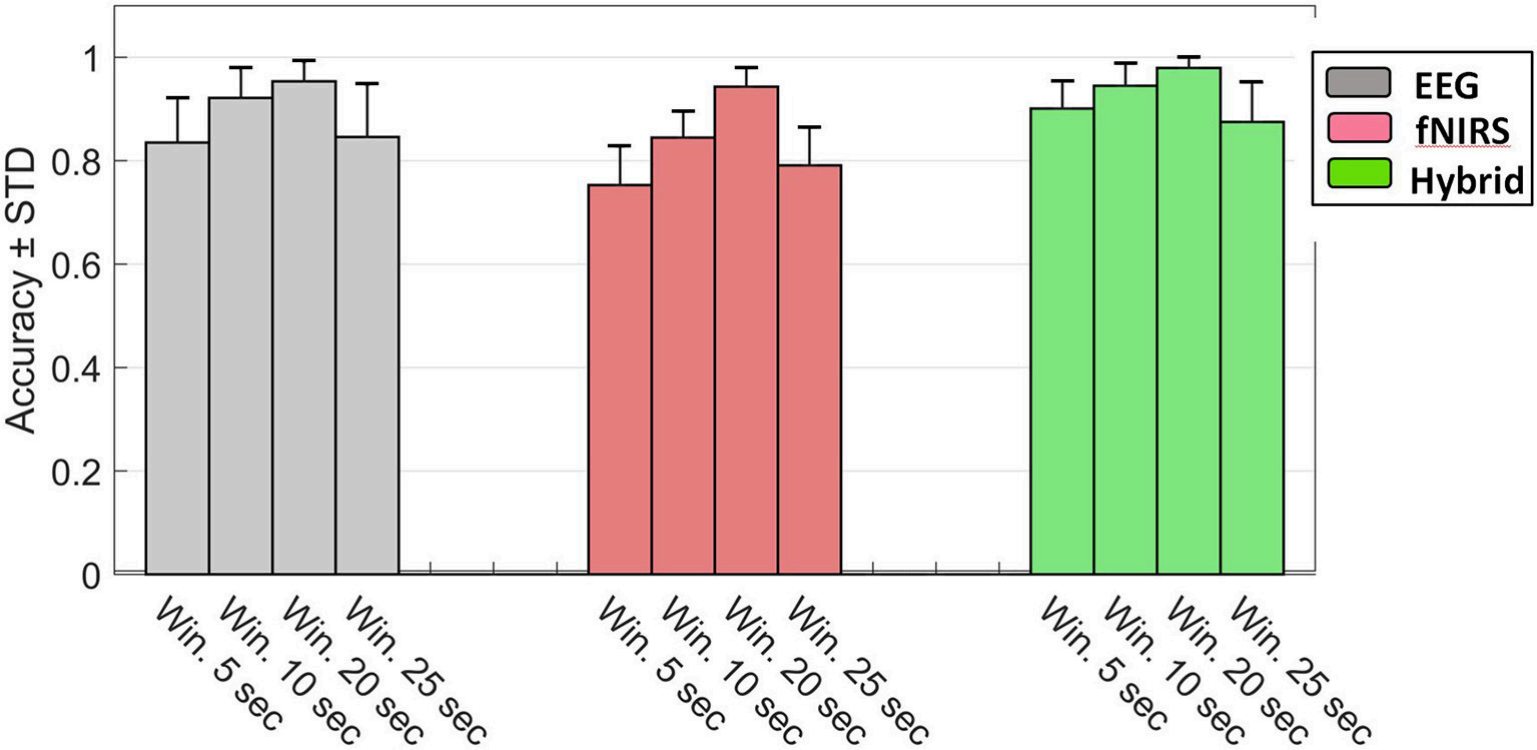
Accuracy of the rest v 3-back classification as a function of window size for EEG (gray), fNIRS (red), and Hybrid (green) systems. Error bars indicate the standard deviation of inter-subject variability.

The foregoing results corresponded to 5 s windows but qualitatively agreed with patterns we observed with other window sizes as well. We also assessed the effect of window length on classification accuracy for EEG, fNIRS, and Hybrid systems. Figure 12 shows the results of this assessment. We examined four different lengths for the windows (5, 10, 20, and 25 s). Change of window length has the same effect on all three types of systems. By increasing the length from 5 to 20 the accuracy increases and declines thereafter.

## Discussion and conclusion

The functional activity of the human brain can be observed with various imaging techniques including fMRI, fNIRS, and EEG. Each of these modalities has its advantages and disadvantages. The advantage of using Hybrid EEG+fNIRS system can be divided into two main categories: First, each of these modalities is measuring the changes in a specific brain physiology. EEG results directly from the electrical activity of cortical and subcortical neurons with a sub-millisecond temporal resolution. On the other hand, fNIRS yields local measures of changes in HbO and HbR concentration and is, therefore, an indicator of metabolic/hemodynamic changes associated with neural activity. Second, the physics of measurement behind EEG and fNIRS are quite different. This property, for example, makes EEG signal prone to blink and muscle artifacts, while this is not the case for fNIRS. Hence using a multimodal recording system we are able to assess brain behavior from different physiological perspectives in addition to compensating for some weaknesses of one modality by the other one.

Our results suggest that EEG+fNIRS combined with a classifier are capable of robustly discriminating among various levels of MWL. In our study, the Hybrid system had an accuracy higher than either EEG or fNIRS alone for every subject. The pooling of EEG and fNIRS features and the inclusion of neurovascular features resulted in a synergistic enhancement, rather than in a diluting effect (which would have given a performance intermediate between the two modalities). In mission-critical contexts such as aviation or surgery, even small improvements in MWL detection can translate into significant gains in safety and efficiency. Our experiments were designed to use WM load (adjusted through the value of *n* in the *n*-back task) as a correlate of MWL in general. Furthermore, EEG and fNIRS can be integrated without excessive cost, effort, or intrusiveness for the user. The combination of all these considerations suggests that EEG+fNIRS should be preferred to only EEG or fNIRS, in developing passive BCIs and other applications which need to monitor users' MWL.

Our preliminary analysis of the experimental data was consistent with expectations. For example Figure 7 indicated that the fraction of accurate responses declined more steeply as *n* increased. This can be explained by noting that temporal tagging is the cognitive process that imposes the greatest load in the *n*-back task, as compared to the other processes which are also involved such as encoding, storage, matching, and inhibition to dampen the oldest memory traces (Jonides et al., 1997).

Temporal tagging, unused in the 0- and 1-back, begins to affect MWL substantially as *n* > 1. Another interesting preliminary result was the observation that HbO showed an anticipatory increase near the end of the rest sessions (Figure 8E). This is consistent with related fMRI findings (Sakai and Passingham, 2003) and with the fact that the PFC is involved in planning future action. A negative correlation between HbO and HbR has been seen in the literature. Based on (Figures 8A–E), the shaded area, which is the standard deviation of normalized HbO and HbR variations within the block for all of the subjects and all of the fNIRS channels, is relatively high. This shows the high level of inter-subjects variability and that might be the reason that we are not seeing such anti-correlated pattern between HbO and HbR in Figure 8. Izzetoglu et al. (2004) showed that the reason behind the drop of the peak of HbO in Figure 8d is that when a participant reaches his maximum performance capacity or in another word starts to overloads cognitively, he loses his concentration on the task and as a result performance as well as the oxygenated hemoglobin changes decline.

Figure 5 did not show any differences between rest and task states that were obvious to visual inspection of the preprocessed EEG or fNIRS signals. Subject and block averaging of various features shown in Figure 9 did, however, indicate that such systematic variations existed. Lower values of the EEG alpha band power in the 2- and 3-back task, and higher values of HbO in the beginning of the task period in the *n*-back (increasing with *n*) relative to those in the rest state were examples of such visible variations. To take advantage of such variations we employed discrimination through linear SVM. In the case of non-linear SVM, the kernel can help with the non-linearly separable data and map it into a new feature space in which the dataset are separable with a linear SVM. In non-linear SVM in order to improve the accuracy choosing the optimum kernel parameters, is necessary. This can reduce classifier's generalization potential for new subjects when we don't want to adjust the kernel's parameters. It will increase the probability of overfitting occurrence, since increasing the complexity level of a classifier gives it the flexibility to match exactly to the train set. In addition, the option of choosing a non-linear SVM depends on the exact application to make a trade-off between a slightly higher accuracy rather than calculation speed. The same trade-off we face to choose the windows size. The results shown in Figure 10, Tables 2, 3 were highly promising for accurately discriminating among the rest and task states. As Tables 2, 3 show, the subject averaged accuracy of the Hybrid system in binary discrimination was lowest (87.2%) for 1-back v rest and highest (96.6%) for 3-back v 0-back. The corresponding lowest and highest results for uni-modal systems were fNIRS (71.6%) and EEG (92.0%), both for 3-back v rest. We calculated the overall average of accuracy one time for all of the binary cases and one time for all of the muli-class cases. We did this calculation for EEG, fNIRS, and Hybrid systems separately. The results show that EEG, fNIRS, and Hybrid system, in the case of binary classification, have 85.9, 74.8, and 90.9% overall accuracy, respectively. EEG, fNIRS, and Hybrid system, in the case of multi-class classification, have 79.6, 57.0, 86.2% overall accuracy, respectively. These numbers convey that the accuracy of each one of EEG, fNIRS, and Hybrid systems are higher for the binary cases. The multi-class accuracies were generally lower; however, note that the chance level accuracy for multi-class classification is less than binary classification (33% for 3-back v 2-back v 1-back, 25% for 3-back v 2-back. v 1-back v rest and also 3-back v 2-back. v 1-back v 0-back, and 20% for 3-back v 2-back. v 1-back v 0-back v rest).

Table 4 reveals that, for all of the four extracted metrics from the confusion matrix (sensitivity, specificity, PPV, NPV), always Hybrid system has a higher value than EEG system and EEG system has a higher value than fNIRS system. In the cases of binary classification, for those that we are detecting between task and rest, the sensitivity of detection of rest state is significantly higher than the sensitivity of detection of task state. As its complement, the specificity of detecting task state is higher than detecting the rest state. In the cases of binary classification, for those that we are detecting between task and task, the sensitivity of detecting the task with a higher difficulty level is more than those with less level of difficulty, although the difference is not very significant. The PPV and NPV are usually more important than sensitivity and specificity. Patients and doctors want to know whether this particular patient is ill rather than whether the test can recognize ill people (Beleites et al., 2013). Here, our result (Table 4) shows that Hybrid system has at the same time a very promising PPV and NPV for all of the classification cases. Except for the case of {1-back v rest}, the minimum of sensitivity, specificity, PPV, NPV for the Hybrid system are 81.4, 82.5, 81.8, and 84.6%, respectively.

Selecting an optimal subset from the full set of features is crucial for achieving high accuracy and avoiding over-fitting. In some applications, e.g., those involving on-board real-time analysis, it may also be important to keep the system size small and avoid computational delays. Figure 11b shows the cumulative sum of *R*^2^ v number of features for three systems which qualitatively agrees with Figure 11a, suggesting that *R*^2^ ranking is an effective method of feature selection. We have not used an explicit artifact rejection step in our analysis. However, it is well known that PCA can segregate non-cerebral artifacts (typically of higher amplitude than contributions of cortical origin) into distinct PCs. Our feature selection based on *R*^2^ then assigns a lower rank to such PCs and they are excluded from a truncated system.

One of the main considerations in developing an online system is computational speed. It is instructive to review the computational loads of particular feature types in conjunction with how effectively they discriminate among rest and task states. For example, Figure 10 shows that PAC is the least discriminating EEG feature. This may be important in designing a compact and efficient detector, as PAC is also the most computationally time-consuming feature. By contrast, the most effective EEG feature (PSD) was also the fastest to compute. In our study, the central processing unit (CPU) time required for computing PSD, PLV, PAC, and Asym_PSD were, respectively, 0.1, 14.3, 44.4, and 0.2 s. The CPU times required for other features were as follows: HbO/R Amp. and HbO-HbR Corr. (0.1 s), HbO/R slope (14.3 s), Std., Skew. and Kurt. collectively (3.3 s), and NVO and NVR (3.4 s).

Our results suggest that Hybrid outperforms the uni-modal systems for each subject (Tables 2, 3), every classification problem (Tables 2, 3), every number of features (Figure 11), and every window size (Figure 12). This could have been due to the neurovascular features that the uni-modal systems do not contain. NV obviously had a higher classification performance rather than any of fNIRS based feature subgroups. However, Figure 10 indicates that such features contribute little if any to the accuracy (two rightmost bars) after the other EEG and fNIRS features have been pooled. The likely explanation instead is related to inter-subject variability. We have found that the top ranked (in terms of *R*^2^) features tend to differ among different subjects. Although, the EEG frequency band power (especially in the alpha range) tended to play an important role for most subjects, for other subjects other feature types, fNIRS- or Hybrid based, dominated the top ranks. An example of this is provided in Table 1 where the third subject's most discriminating PCs were neurovascular. EEG and fNIRS use different physical processes for detection and the underlying physiology which they detect are different. Hence deficiencies such as artifacts, weak sensor coupling, or subject variability leading to a weak signal would selective affect only one modality rather than both. The Hybrid advantage may be associated primarily with the complementary nature of the individual modalities.

Figure 12 indicates that accuracy can be increased by using larger windows. But this presents a tradeoff between accuracy and rapid detection. The windows with the highest accuracy were 20 s long and may be impractical for some applications if the online response to rapid changes in MWL is desirable. As window size increases, although the amount of information per window likely increases, the number of windows available for training the classifier decreases. Fewer training data are expected to cause the classifiers to underperform (Grimes et al., 2008). The decline in accuracy in Figure 12 for windows >20 s may be due to the excessively small number of data available for training.

A handful of studies on concurrent EEG and fNIRS and WM tasks have been previously published. Hirshfield et al. (2009) combined an 8 channel fNIRS covering the forehead with 32 channel whole-head EEG with *N* = 4 subjects as they performed a counting and mental arithmetic task with adjustable difficulty. They used separate classifiers for the fNIRS and EEG (*k*-nearest neighbor and Naive Bayes classification, respectively) and obtained a maximum accuracies of 64% (with fNIRS) and 82% (EEG). They did not attempt to use the multi-modal data concurrently. The generally higher accuracy of the EEG is consistent with our results. Their overall lower accuracies relative to our results may be due to the relatively short 2 s feature extraction window. Liu Y. et al. (2013) used a 16-optode fNIRS system covering the forehead and 28 EEG sensors at the standard 10–20 sites, with *N* = 16 subjects performing a *n*-back task. They found significant correlations between WM load and some EEG frequency band powers as well as HbO and HbR, however did not attempt classification. Their study focused on discovering neural correlates of the effects of practice time on performance. Coffey et al. (2012) recorded three fNIRS channels over the left forehead together with 8 EEG electrodes placed mainly in the frontal and central areas, from *N* = 10 subjects in a *n*-back task. They extracted EEG frequency band power and fNIRS Hb amplitude features from 5 s windows and employed them in linear discriminant analysis classifiers. They report maximum accuracies of 89.6% (EEG), 79.7% (fNIRS), and 91.0% (Hybrid). Their results differed from ours in that in some subjects all their systems had very low accuracies and their Hybrid accuracies were not always higher than those of both uni-modal systems. However, the fact that EEG generally had the higher uni-modal accuracy and that Hybrid could attain the highest observed accuracy were consistent with our findings. The differences from our results could be attributed to the relatively lower number of sensors and fewer types of features they employed.

Acquiring the very low-frequency (VLF) oscillations (< 0.5 Hz) in the EEG signal requires highly specialized amplifiers (DC-coupled, high input impedance, high DC stability, and a wide dynamic range; Demanuele et al., 2007). In addition, VLF oscillations are known to be linked with specific pathologies such as epileptic seizures or attention deficit hyperactivity disorder (Steriade et al., 1993; Vanhatalo et al., 2004; Demanuele et al., 2007) that are not within the range of interest in this study. On the other hand, some studies (Gevins et al., 1997; Berka et al., 2007; So et al., 2017) demonstrated EEG within gamma range as a biomarker for discrimination between different cognitive states. We defined the band-pass filter cutoff frequency (0.5–80 Hz) based on these criteria. Although in the feature extraction section, we did not consider gamma frequency range features and have considered this as the future work.

The present study had several limitations which we have not directly addressed due to constraints of available time or effort. Firstly, the group of subjects included only one female. This may have been due to the demographics of the subjects, who happened to be interested in volunteering for our study. In addition to the neural correlates of MWL, we have recorded the subjects' performance characteristics. However, it may prove insightful to collect data on the MWL by using additional techniques such as self-reporting, which was not done in this study. In some studies for assessment of MWL participants filled out the NASA Task Load Index (NASA TLX) questionnaire (Hart and Staveland, 1988) to provide a subjective evaluation of the mental demand induced by different levels of task difficulty. In this study, we implicitly used the assumption that an increase in the level of task difficulty will result in a higher MWL. This can be also considered in future studies. In addition, it is possible that during the course of an experiment the subjects' performance and MWL change through training effects. Studying the performance and neural correlates of MWL for subsets of our data could reveal differences in the beginning and at the end of the study. This would also require an additional investigation of statistical validity, and was not attempted. The statistical significance of the results of our study was demonstrated through a two-way ANOVA that showed significant differences in the accuracy of the Hybrid v uni-modal systems. However, we have not investigated whether a smaller group of subjects would still yield a significant result. We have investigated the capabilities of various subsets of the types of features that were available. It would also be illuminating to investigate the classification accuracy of subsets of the full array of our sensors. Such information can help design more compact headsets and is the subject of an ongoing study. The headset we used is lightweight and no discomfort was reported by any of the subjects. However, wearing it may nevertheless affect performance, and this could be revealed in a parallel set of experiments which we have not done. The primary goal of our study was to apply machine learning techniques in discriminating levels of MWL. We used multiple statistical techniques to ensure that the statistical significance of the values of accuracy that we obtained for such discrimination. Our observations regarding the range of changes of Hb are therefore only qualitative and observational, serving to ensure that our results are consistent with expectations.

In this study, we have taken steps toward investigating the EEG+fNIRS feature extraction and analysis methods by using a popular WM task. We anticipate and hope that converging efforts in Hybrid hardware integration (Safaie et al., 2013) and data analysis (Biessmann et al., 2011; Keles et al., 2016), potentially based on detailed knowledge of underlying physiology (Bari et al., 2012; Mandrick et al., 2016a), will lead to more effective passive BMIs and other applications in neuroergonomics.

## Author contributions

HA and AO designed the study. HA collected and analyzed the data. HA, AO, and MG wrote the manuscript.

### Conflict of interest statement

AO participated in the development of the wireless portable EEG device (microEEG), which was used in this research. He holds a financial interest in Bio-Signal Group which is the maker of microEEG. The other authors declare that the research was conducted in the absence of any commercial or financial relationships that could be construed as a potential conflict of interest.
